# Climate Based Predictability of Oil Palm Tree Yield in Malaysia

**DOI:** 10.1038/s41598-018-20298-0

**Published:** 2018-02-02

**Authors:** Pascal Oettli, Swadhin K. Behera, Toshio Yamagata

**Affiliations:** Japan Agency for Marine-Earth Science and Technology, Application Laboratory, Yokohama, 236-0001 Japan

## Abstract

The influence of local conditions and remote climate modes on the interannual variability of oil palm fresh fruit bunches (FFB) total yields in Malaysia and two major regions (Peninsular Malaysia and Sabah/Sarawak) is explored. On a country scale, the state of sea-surface temperatures (SST) in the tropical Pacific Ocean during the previous boreal winter is found to influence the regional climate. When El Niño occurs in the Pacific Ocean, rainfall in Malaysia reduces but air temperature increases, generating a high level of water stress for palm trees. As a result, the yearly production of FFB becomes lower than that of a normal year since the water stress during the boreal spring has an important impact on the total annual yields of FFB. Conversely, La Niña sets favorable conditions for palm trees to produce more FFB by reducing chances of water stress risk. The region of the Leeuwin current also seems to play a secondary role through the Ningaloo Niño/ Niña in the interannual variability of FFB yields. Based on these findings, a linear model is constructed and its ability to reproduce the interannual signal is assessed. This model has shown some skills in predicting the total FFB yield.

## Introduction

The production of vegetable oils is globally expanding at a rate of 4.5% a year (period 2004–2013)^1^. This augmentation is a response to the increasing demand in food industry and household cooking for edible oil, cosmetic industry, and biodiesel fuel. In particular, vegetable oils are generally considered as a credible alternative to fossil fuels^2^ that may contribute to sustainable power supply^3–6^. But the rapid expansion of oil crops in the world is also seen as an important threat for the local environment^7–10^; for example, soil and water pollution, deforestation, aquifer depletion, increase of land price, and even serious concurrence between oil crops and food crops.

Among major vegetative oils, the palm oil is the most widely produced, representing 37% of the total production of vegetable oils in 2013^1^ with an annual growth rate of 6.7% for the period 2004–2013^1^. Its production is five times greater per unit of land than any other oil crop^11^; it is a highly profitable source of income through exports from tropical areas^12,13^. Over the period 2004–2013, on average, 88.1% of crude palm oil (CPO) were produced in Asia^1^, Indonesia and Malaysia contributing the most, accounting for 81.7% on average of the total global production^1^. In Malaysia, the oil palm industry is an important contributor to the gross national income, accounting for USD 16.8 billion (2011) and employing more than 600,000 people^14^.

However, the CPO price is dependent strongly on economic, political and environmental factors^15^. Thus, the supply and demand of palm oil is highly related to the economy of importers. In the context of healthy economy in these countries, the CPO price is likely to rise. Also, the price of competing vegetable oils is affecting the CPO price. For example, less production of soybean will tend to increase price of soybean oils. To compensate for the gap in supply of vegetable oils, the demand on CPO will also increase, helping to escalate CPO price. Other factors are the import policies of importing countries (in the context of boycott of oil palm-based products) and the changes in taxation and import duty (in the context of protectionism). Finally, the weather patterns are also affecting the CPO price. Thus, dry spell is likely to reduce the production of CPO, as most of cropping systems are rainfed^16^.

The interannual variability of rainfall in Malaysia is controlled by the interannual variation of sea-surface temperature (SST) anomalies in the tropical Pacific, i.e. the El Niño/Southern Oscillation (ENSO)^17–19^. During boreal winter, regions of the Maritime Continent located north of the equator tend to have more anomalies in precipitation related to ENSO^20^, particularly northern Borneo in the case of Malaysia^21^. Its impact on the palm oil is also known well, and is considered as the main factor explaining the interannual variability of FFB yields and CPO production^22–25^. Thus, yields in the year following an El Niño event tend to be less than that on average, while after a La Niña event, it tends to be more than the average.

Another source of the interannual variability of rainfall is the Indian Ocean Dipole (IOD)^26,27^, with negative (positive) rainfall anomalies appearing during a positive (negative) dipole event in boreal fall, i.e. during the peak season of IOD^28^. Recently discovered El Niño Modoki^29^ also favors a drier condition in both Borneo and Peninsular regions^21^. In addition, rainfall in Malaysia is influenced by the Northeast Monsoon or winter monsoon^30,31^ (while strongly related to ENSO^18,20^), the Madden-Julian Oscillation^27^ and the Borneo vortex^27,32^. Both El Niño and positive IOD tend to favor haze due to forest fires^33,34^, which, in turn, affects the production of CPO by degrading the radiation conditions^35^.

We first focus on the description of the interannual variability of the production of fresh fruit bunches on a country scale and for two main geographic entities of Malaysia, the peninsula (hereafter Peninsular Malaysia) in the West and the provinces in the Northern part of the Borneo island (hereafter Sabah/Sarawak) in the East. The influence of local variables, such as rainfall, temperature or solar radiation, on the productivity of palm trees is investigated. We then emphasize the role of the remote climate modes on the modulation of the local conditions. Finally, statistical models explaining the annual yields for the whole Malaysia and for the two regions in the country are developed and their forecast ability is evaluated.

## Results and Discussion

In Malaysia, FFB is produced throughout the year^36^, but it is marked by a strong seasonality due to variations in reproductive growth^37,38^; the yield is more in boreal fall compared to boreal spring^39,40^. Because of the diversity in the climate and geographic conditions, we have divided the country into two regions. The yields in both geographical areas are locked to seasons^39,40^, with a minimum in production around February and a peak in September-October. In recent years, the eastern states of Sabah (the Malaysia’s biggest palm-growing state) and Sarawak give slightly larger average annual yields than Peninsular Malaysia^1,41^.

**Figure 1:**
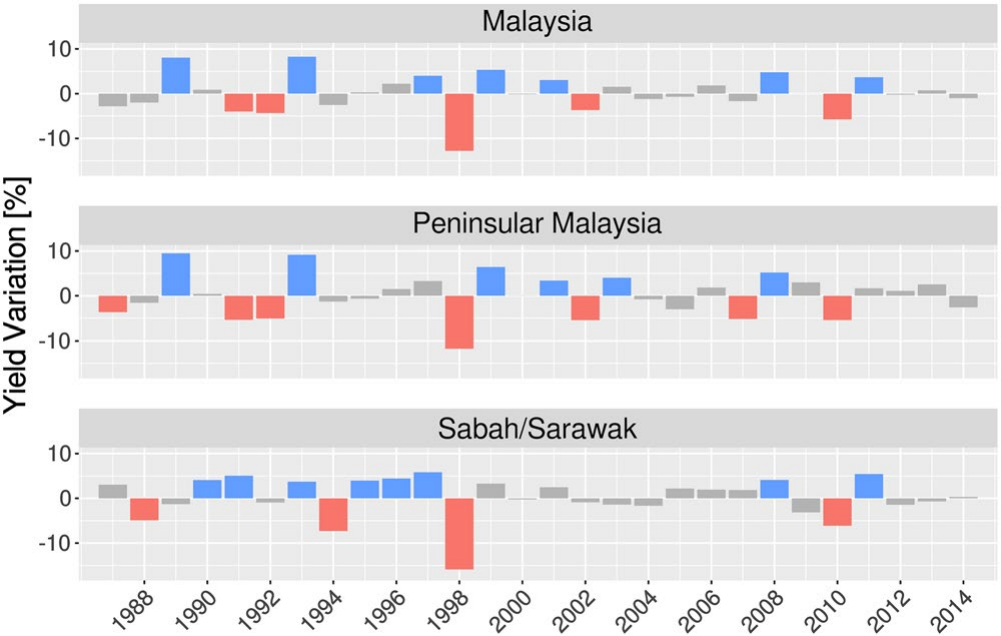
Oil palm fresh fruit bunch (FFB) yields interannual variability in Malaysia (top panel), Peninsular Malaysia (middle panel) and Sabah/Sarawak (bottom panel). The figure was generated by R software version 3.4.1 (https://www.R-project.org/) with package “*ggplot2*” version 2.2.1.

The interannual variation^42^ of yearly FFB yields^41^ (aggregated from January to December) is shown in Fig. 1 for Malaysia (upper panel), Peninsular Malaysia (middle panel) and Sabah/Sarawak (lower panel). A strong variability in yearly yields is noticed for all regions, with marked interannual rises and drops during the 28 years of the study period. Based on those interannual variability, we define years of low (high) FFB production when the negative (positive) percentage is larger than −0.7 (0.7) standard-deviation (i.e. ±3.02% for Malaysia, ±3.35% for Peninsular Malaysia and ±3.29% for Sabah/Sarawak), respectively. This threshold is chosen because the size of the sample is not enough to work with a ±1 standard deviation. In this way, five years of low FFB yields (1991, 1992, 1998, 2002, 2010) and seven years of high FFB yields (1989, 1993, 1997, 1999, 2001, 2008, 2011) are picked for Malaysia. Similarly, seven years of low yields (1987, 1991, 1992, 1998, 2002, 2007, 2010) and six years of high yields (1989, 1993, 1999, 2001, 2003, 2008) are picked for Peninsular Malaysia, and four years of low yields (1988,1994,1998,2010) and eight years of high yields (1990, 1991, 1993, 1995, 1996, 1997, 2008, 2011) are picked for Sabah/Sarawak. All other remaining years are considered as normal for respective regions.

### Local atmospheric variables

Best possible FFB yields are obtained under optimal climatic conditions^43^, with at least 2,000 mm of rainfall homogeneously distributed throughout the year corresponding to around 167 mm month^−1^. Also, minimum temperatures should be between 22 and 24 °C and maximum temperatures between 29 and 33 °C, while relative humidity should be greater than 85%. Finally, solar radiation should be at least 16 or 17 MJ m^−1^ d^−1^. Composites of rainfall, minimum and maximum temperatures at 2-m height, surface net solar radiation, relative humidity at 2-m and potential evapotranspiration are constructed following identified years of lower and higher FFB production. Monthly spatial mean for each group of years are calculated between December, of the year of harvest, and January, three years before, i.e. for a span of 48 months. This period covers different stages of palm tree cycle^38,44,45^, particularly the different stress-sensitive periods^36,45^; sex determination (31 to 20 months before harvesting year), inflorescence abortions (12 to 8 months) and bunch failures (4 to 2 months).

**Figure 2:**
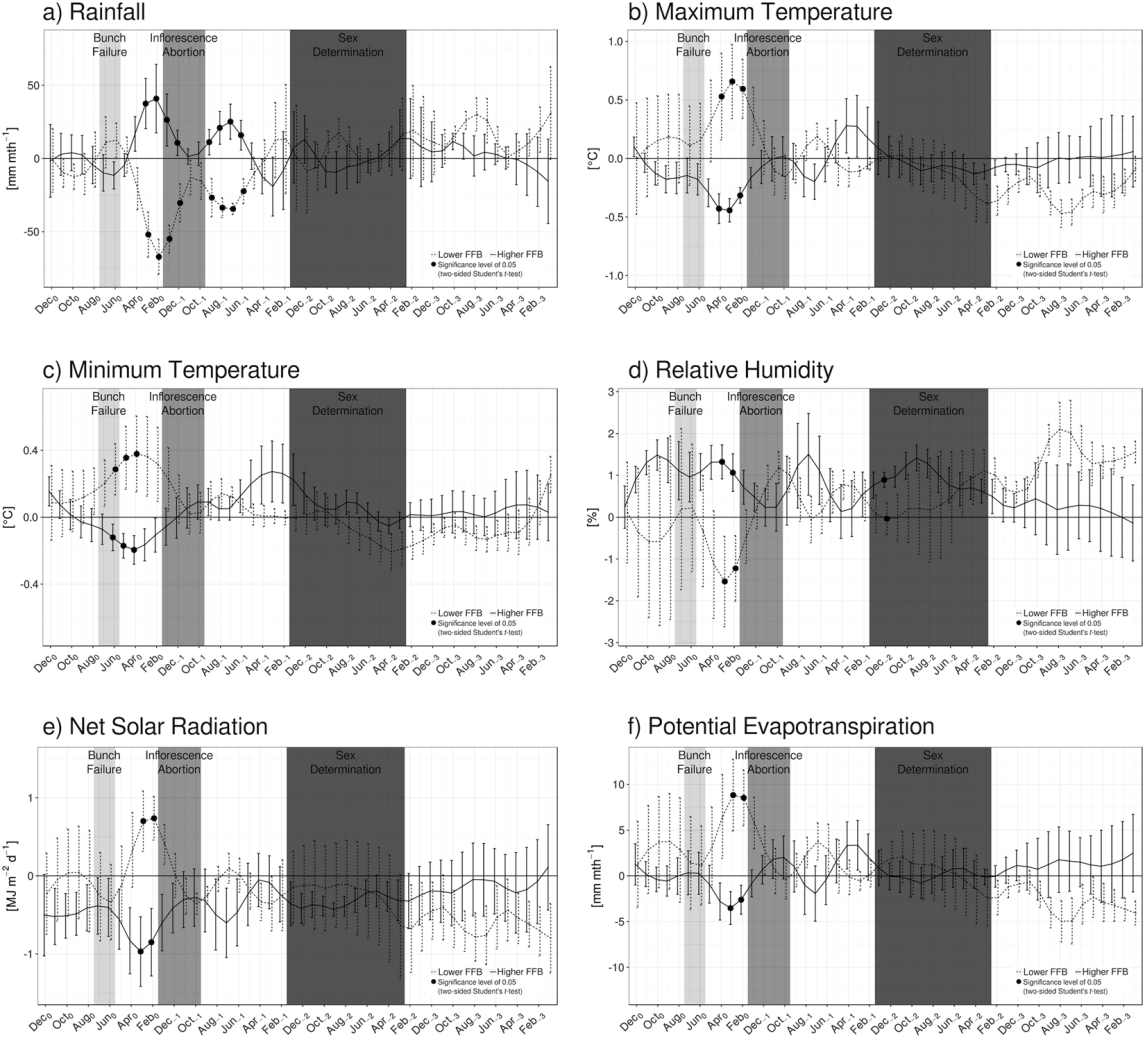
Composite analysis of deviations from the mean for (**a**) rainfall, (**b**) maximum temperature at 2-m height, (**c**) minimum temperature at 2-m height, (**d**) relative humidity at 2-m height, (**e**) solar radiation and (**f**) potential evapotranspiration for Malaysia. Vertical bars denote the standard error of the mean. Shaded areas denote stress-sensitive periods considering the peak of fruits harvesting in September-October, i.e. sex determination (dark grey), inflorescence abortion (grey) and bunch failure (light grey). Significant differences between means at 95% level (according to two-sided Student’s *t*-test with 10,000 permutations) are showed by black dots. The figure was generated by R software version 3.4.1 (https://www.R-project.org/) with package “*ggplot2*” version 2.2.1.

Composite time series for the whole Malaysia are shown in Fig. 2. At the country scale, the period covering from late boreal winter to early boreal spring is a key to discriminate years of lower production from years of higher one, potentially providing useful information to estimate the total yields at the end of the year. Considering the peak season of FFB harvesting to be September-October^40^, this coincides with the period of inflorescence abortion (Fig. 2a–f, grey area). Between January (Jan_0_) and March (Mar_0_), of low FFB production years, Malaysia is anomalously dry with rainfall about 60 mm month^−1^ below average (Fig. 2a, black dashed line) and a level of relative humidity less than normal (Fig. 2d, black dashed line). Associated with an anomalously enhanced potential evapotranspiration (Fig. 2f, black dashed line), this indicates drought condition at the country scale is a yield-reducing factor^38,44–46^. This water stress is also explainable by the anomalously warmer maximum and minimum temperatures (Fig. 2b,c, black dashed line) and the stronger surface net solar radiation (Fig. 2e, black dashed line), which tend to favor the evaporation. It might be counterintuitive that more than normal radiation is associated with less yields, knowing the positive effect of radiation on photosynthesis. However, we believe the monthly time scale is not the best one to capture the impact of solar radiation on fruit growth. On that scale, the effect of solar radiation is reduced by the importance of water stress. On the other hand, better FFB production is favored by wetter condition: more than normal precipitation (Fig. 2a, black solid line), higher relative humidity (Fig. 2d, black solid line) and less evapotranspiration (Fig. 2f, black solid line) in late boreal winter and late spring. We note that there is certainly a limit for the concept of “higher rain-higher yield”; it is known that too high continuous rains reduce the population of the weevils pollinators and subsequently the production of the palms. Because of more precipitation and associated cloudy conditions, less radiation can reach the surface (Fig. 2e, black solid line). Again, this appears to be counterintuitive, but the better FFB production is explained by the prominence of water condition over the impact of radiation at a monthly time scale. Also, anomalously cooler temperatures exist (Fig. 2b,c, black solid line) reducing the risk of fruits mortality. No clear differences appear in two others stress-sensitive periods (still considering September-October as the peak of fruits harvesting), i.e. sex determination (Fig. 2a–f, dark grey area) and bunch failure (Fig. 2a–f, light grey area) periods.

Results are somehow similar in Peninsular Malaysia (Supplementary Fig. S1) and Sabah/Sarawak (Supplementary Fig. S2), with similar influences arising from local atmospheric conditions during late boreal winter-early boreal spring: Sabah/Sarawak suffers from less rainfall during El Niño, while Peninsular Malaysia is relatively spared^18,20,21^. For rainfall anomalies as well as evapotranspiration, there is no statistically significant difference between years of lower (black dashed line) and higher (black solid line) FFB. In Sabah/Sarawak, the water stress is a more important limiting factor, considering the significant difference of anomalies in rainfall, relative humidity and potential evapotranspiration. At the spatial and temporal scales of the present interest, solar radiation doesn’t play a decisive role in determining the yearly FFB yields. However, at finer time scales (i.e. hourly or daily), the quantity of energy received by the trees is expected to condition the quality and quantity of fruits production^11,35^.

### Large scale climate modes

**Figure 3:**
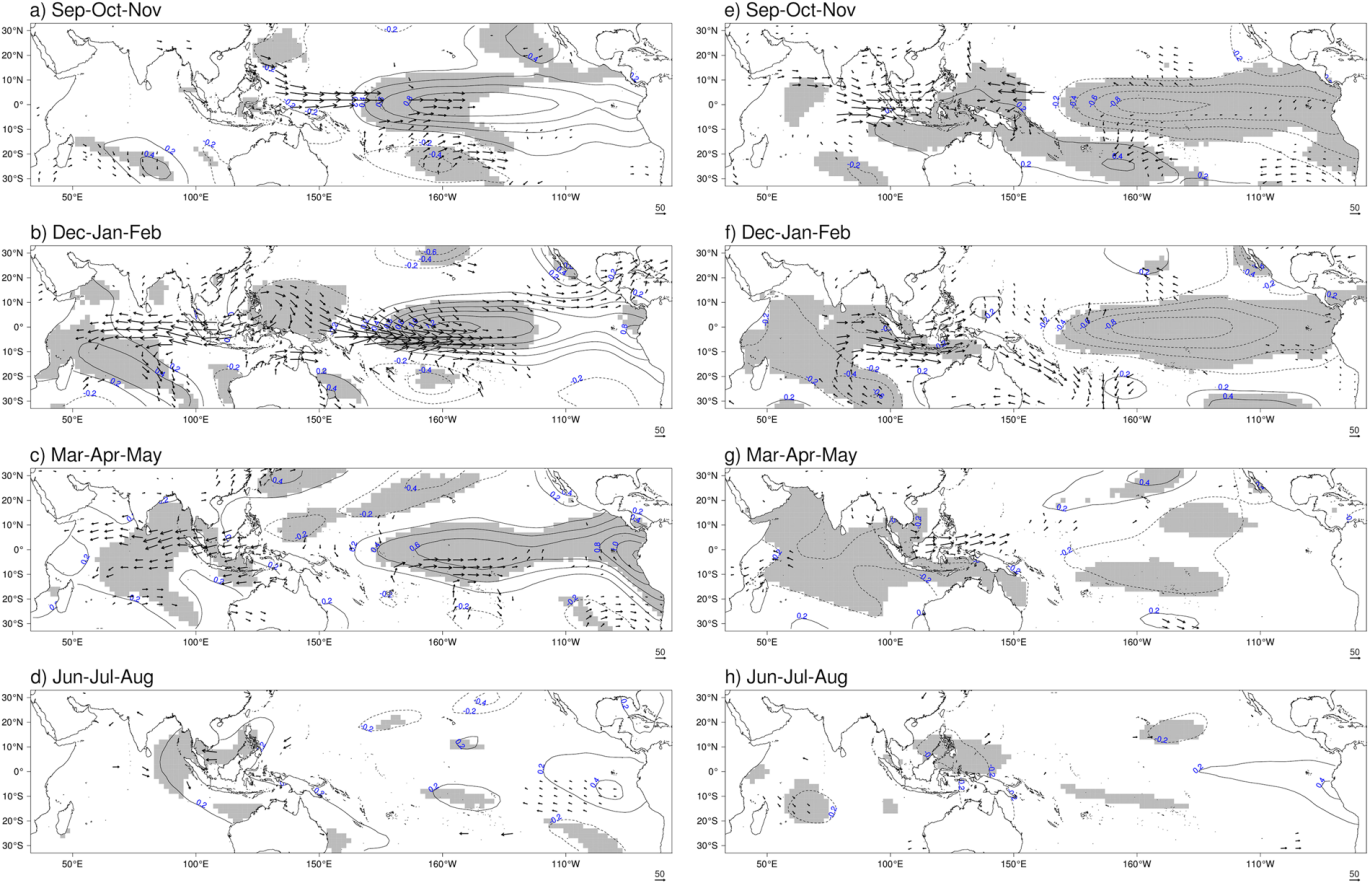
Composites of deviations from the mean for sea-surface temperatures (contour) and humidity flux (vector) anomalies for Malaysia case. Composites are calculated for years of lower FFB (left column) and years of higher FFB (right column). Only humidity flux anomalies significant at the 95% level (according to Hotelling’s T2 test) are plotted. Grey shading denotes SST anomalies significant at the 95% level (according to two-sided Student’s *t*-test). Composites are calculated for SON (**a**,**e**), DJF (**b**,**f**), MAM (**c**,**g**) and JJA (**d**,**h**). The figure was generated by R software version 3.4.1 (https://www.R-project.org/) with package “*rasterVis*” version 0.41.

Composites of sea-surface temperature and vertically integrated moisture flux anomalies are constructed based on the identified years of lower and higher FFB production. Considering the key period previously identified and the life cycle of a typical El Niño event (start in boreal summer, peak in boreal winter and decay during the following spring^47^), four periods are considered here; boreal fall of previous year and subsequent boreal winter, spring, and summer seasons. In the boreal fall i.e. September-October-November (SON) for years of lower FFB yields, positive SST anomalies exist in the equatorial Pacific Ocean (Fig. 3a). Eastward flux anomalies take place between the Maritime Continent and the central Pacific Ocean. At this lead time, no significant signal is seen in the Indian Ocean though it is the peak season of the IOD^27^. For years of higher FFB yields, negative SST anomalies (below −1 °C) appear in the equatorial Pacific Ocean in SON (Fig. 3e), highlighting the positive impact of La Niña on palm oil trees productivity^22,24^. A convergence in the flux anomalies exists near New Guinea (Fig. 3e), as well as an anomalous cyclonic circulation in the southeastern Indian Ocean, generating eastward flux anomalies over the Maritime Continent. The later might be related to the existence of Ningaloo Niño signal off Western Australia^48^. As a consequence, Malaysia and surrounding area receive more humidity, originating from the equatorial Indian Ocean.

In the following winter season, i.e. December-January-February (DJF), positive SST anomalies become stronger in tropical Pacific, particularly in the central Pacific Ocean (Fig. 3b) where anomalies are above 1 °C (significant at 95% confidence level). This result is consistent with the results of previous studies^22–25^ that show negative impact of El Niño on yearly yields. In the vicinity of New Guinea, a divergence in the flux anomalies is visible, as well as an anomalous anticyclonic circulation south of the Philippines over negative SST anomalies. The subsidence over the eastern part of the Maritime Continent is a response to the anomalous heating generated in the equatorial Pacific Ocean. Consequently, the subsidence generates westerly anomalies over the Western Pacific and dry easterly anomalies over the Maritime Continent (therefore over Malaysia) as well as over the equatorial Indian Ocean. This might explain drier conditions in Malaysia, as described by Tangang and Juneng (2004)^18^, leading to unfavorable environment for palm fruits growth. Significant SST anomalies are also noticed off Western Australia that resemble the Ningaloo Niña^48,49^, especially since DJF is the peak season of this coastal phenomenon^40^. The existence of cold SST anomalies in this region may partly explain the anomalous anticyclonic circulation in the southern Indian Ocean^48^, helping the development of the divergence previously described. In this study, we have not separated the effects of locally and non-locally amplified Ningaloo Niña^48^ to distinguish their influences from El Niño as that separation did not affect the results described later. In the opposite case of higher FFB yields, negatives SST anomalies (below −1 °C) persist in the equatorial Pacific Ocean together with the decaying Ningaloo Niño signal in the eastern Indian Ocean (Fig. 3f).

In boreal spring, i.e. March-April-May (MAM), only SST anomalies in the Pacific Ocean and westerly flux anomalies over the Indian Ocean persist (Fig. 3c). Positive anomalies in the Indian Ocean appear after El Niño^50^. The composite of higher FFB yields shows negative SST persisting in MAM in the equatorial Pacific Ocean (Fig. 3g), albeit to a lesser extent than during SON and DJF. Finally, in boreal summer i.e. June-July-August (JJA), only weak positive SST anomalies are seen in the seas surrounding Malaysia (Fig. 3d). During ENSO, more forest fires occur, generating haze, which is also a yield-reducing factor^35,51^ due to reduction in the solar radiation.

**Figure 4:**
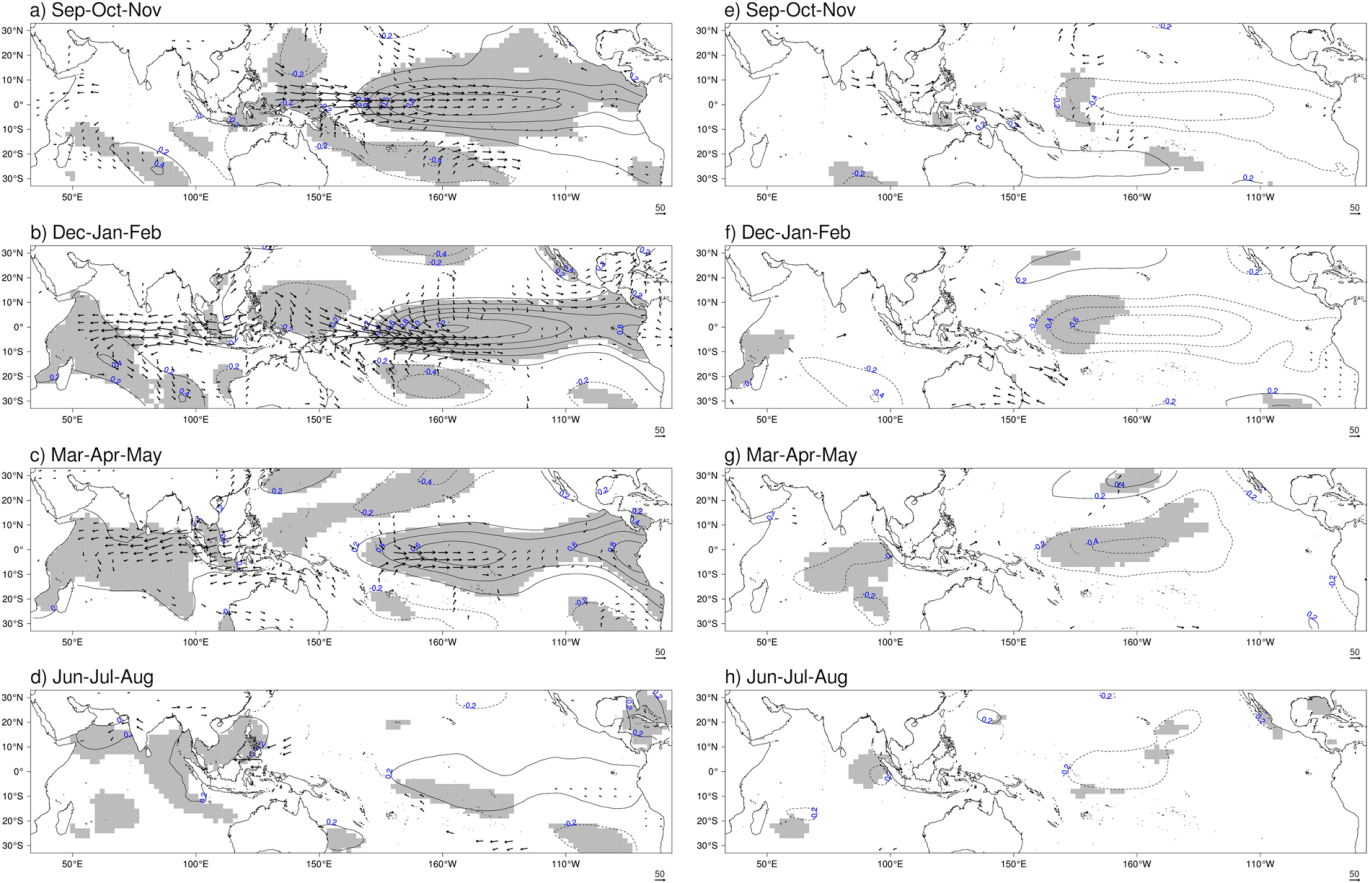
Composites of deviations from the mean for sea-surface temperatures (contour) and humidity flux (vector) anomalies for Peninsular Malaysia case. Composites are calculated for years of lower FFB (left column) and years of higher FFB (right column). Only humidity flux anomalies significant at the 95% level (according to Hotelling’s T2 test) are plotted. Grey shading denotes SST anomalies significant at the 95% level (according to two-sided Student’s *t*-test). Composites are calculated for SON (**a**,**e**), DJF (**b**,**f**), MAM (**c**,**g**) and JJA (**d**,**h**). The figure was generated by R software version 3.4.1 (https://www.R-project.org/) with package “*rasterVis*” version 0.41.

**Figure 5:**
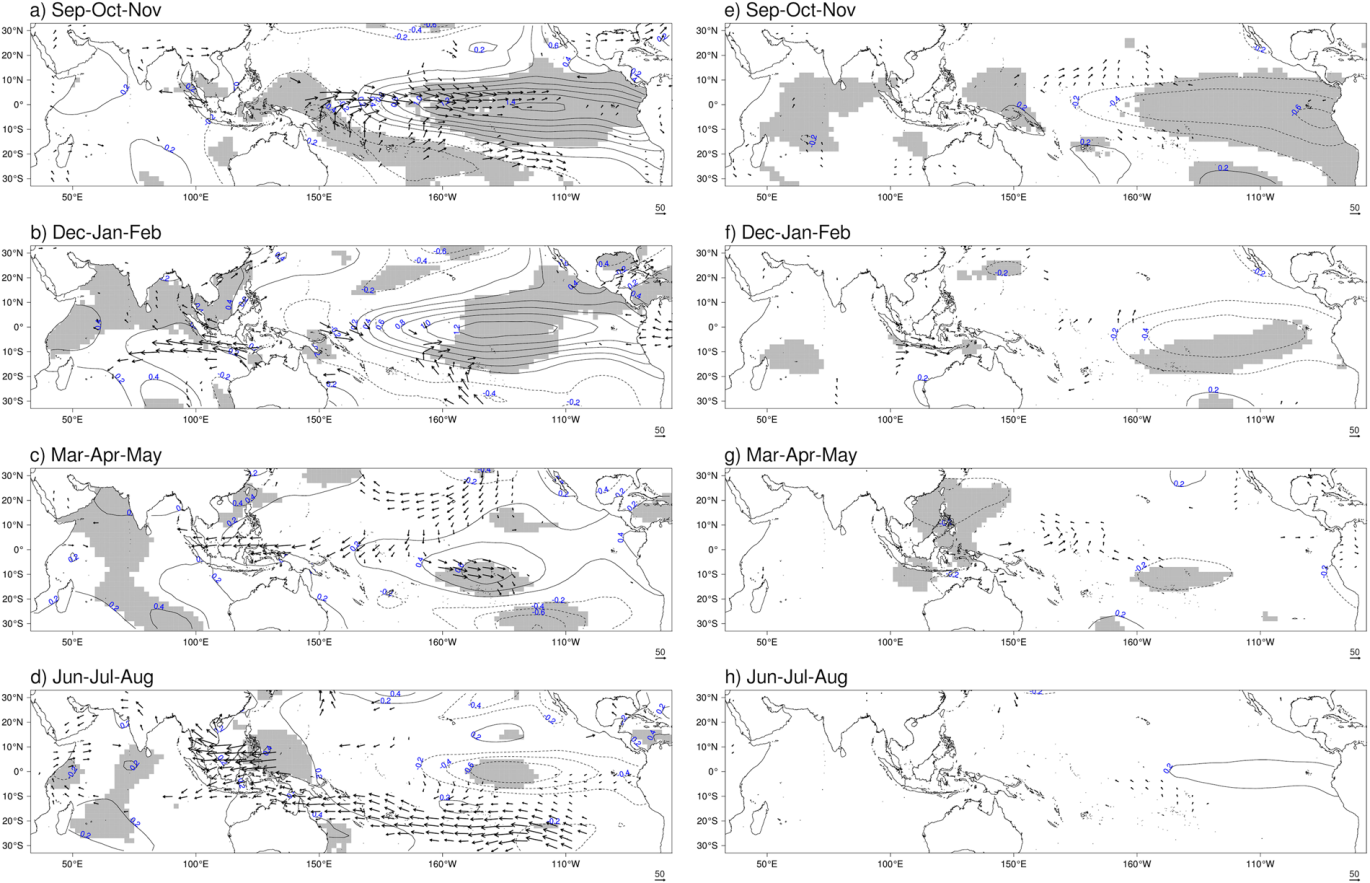
Composites of deviations from the mean for sea-surface temperatures (contour) and humidity flux (vector) anomalies for Sabah/Sarawak case. Composites are calculated for years of lower FFB (left column) and years of higher FFB (right column). Only humidity flux anomalies significant at the 95% level (according to Hotelling’s T2 test) are plotted. Grey shading denotes SST anomalies significant at the 95% level (according to two-sided Student’s *t*-test). Composites are calculated for SON (**a**,**e**), DJF (**b**,**f**), MAM (**c**,**g**) and JJA (**d**,**h**). The figure was generated by R software version 3.4.1 (https://www.R-project.org/) with package “*rasterVis*” version 0.41.

At a regional scale, El Niño and Ningaloo Niña signals, as well as westward moisture flux anomalies over Maritime Continent, are found during years of lower FFB yields in Peninsular Malaysia and in Sabah/Sarawak, with some variation. For Peninsular Malaysia case, the signal in the equatorial Pacific Ocean persists throughout the period of study (Fig. 4a–d), together with the Ningaloo Niña signal in boreal fall and winter (Fig. 4a,b). In Sabah/Sarawak case, a transition between warm SST anomalies, accompanied by Ningaloo Niña (Fig. 5a,b), and cold SST (Fig. 5c,d) anomalies takes place. As a response to the cold anomalies in the equatorial Pacific Ocean, a patch of positive SST anomalies appears east of the Philippines in JJA (Fig. 5d). Westward moisture flux anomalies over Sabah/Sarawak are also noticeable. It is not clear why such a combination would be unfavorable for FFB yields. Perhaps, the moisture flux is not enough to favor higher yields during these events. During years of higher yields, La Niña influence dominates over Peninsular Malaysia (Fig. 4e–g) and Sabah/Sarawak (Fig. 5e–g), again with some variations. In the Peninsular Malaysia case, SST anomalies resemble more of a La Niña Modoki, while SST anomalies in Sabah/Sarawak case show the typical pattern of a canonical La Niña.

### Annual yields, statistical models and tentative forecasts

**Figure 6:**
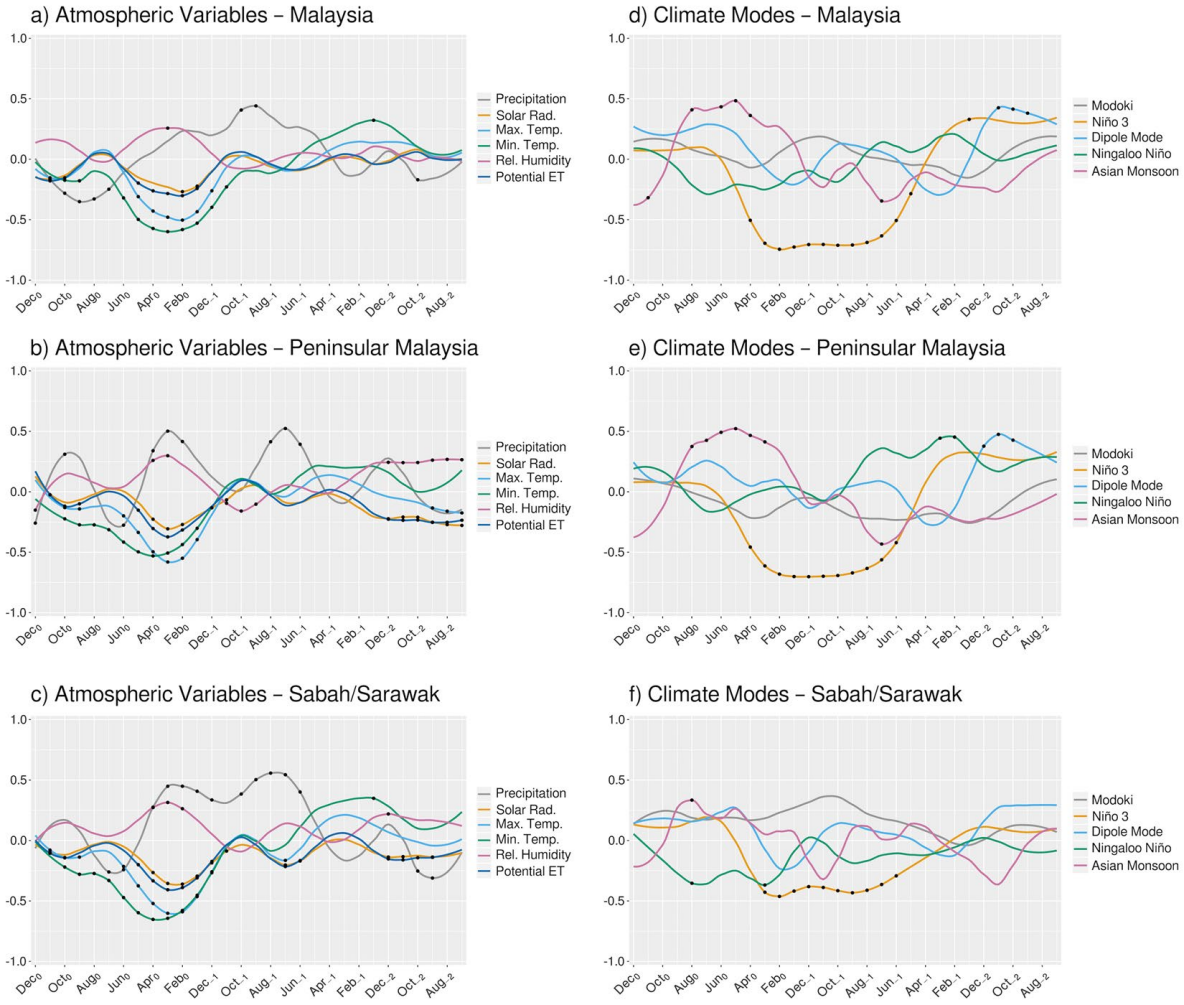
Left column: lagged correlation coefficients between yearly FFB yields and precipitation (grey line), solar radiation (orange line), maximum temperature at 2-m height (sky blue line), minimum temperature at 2-m height (bluish green line), relative humidity at 2-m height (reddish purple line), potential evapotranspiration (blue line). Right column: lagged correlation coefficient between yearly FFB yields and ENSO (orange line), and lagged partial correlation coefficients between yearly FFB yields and Modoki (grey line), Indian Ocean Dipole Mode (sky blue line), Ningaloo Niño (bluish green line), Asian Monsoon (reddish purple). Calculations are done for Malaysia (top row), Peninsular Malaysia (middle row) and Sabah/Sarawak (bottom row). Black dots denote correlation coefficients significant at the 95% level (according to a random-phase test). The figure was generated by R software version 3.4.1 (https://www.R-project.org/) with package “*ggplot2*” version 2.2.1.

Annual yields in different geographic entities are influenced by both local atmospheric variables and large-scale climate modes. Since late 1940’s, different statistical models have been built to forecast the yields of fresh fruit bunches using climate data, for West Africa^38^, as well as Malaysia^25,52,53^. In particular, Foong (1982)^52^ showed an effect of temperature on monthly yields and the ability of the model for a 3-month lead forecast based on atmospheric temperatures. More recently, remote influence of climate modes, such as ENSO^22,54^, has been taken into account to model yields in Malaysia. Here, we evaluate a statistical model combining the effects of both local climate data and large-scale modes of climate variation. First, lagged correlations are calculated among annual FFB yields and local atmospheric parameters (Fig. 6a–c). Partial lagged correlations^55^ are also calculated between yearly yields and large-scale climate indices to elucidate a sole effect of each climate mode under the assumption of independence among climate modes (Fig. 6e–g). In this analysis, atmosphere and ocean parameters are leading yields for 30 months (maximum lag) to 0 (concurrent correlation). For whole Malaysia, strongest relationships among atmospheric parameters and palm yields are found in late boreal fall and boreal spring (Fig. 6a). This result is consistent with the composite analysis, where the differentiation between years of lower and higher FFB yields is the most pronounced during those seasons. Negative correlations between annual yields and temperatures show the negative impact of anomalously high temperatures on yields. And a positive correlation between yields and precipitation in spring agrees well with the known benefit of higher rainfall on fruits production. Here we note that water stress is mainly visible through the rainfall. Relative humidity and potential evaporation play a minor role on the interannual variability of FFB yields but it doesn’t mean their effects are negligible at other temporal and/or spatial scales, which we are not discussing here.

Rainfall impact is even more important in summer time, the year before the fruits harvest. As already shown by the composite analysis, ENSO is a key factor to explain the interannual variability of FFB yields in Malaysia (Fig. 6d). Negative correlation between yields and Niño3 index is consistent with lower harvest following an El Niño event. Similar results are found for the Peninsular Malaysia, with the February-April period as a key season. Interannual variability in yields of this region is mainly explained by variations in temperature and rainfall (Fig. 6b). Lower (higher) yields are due to higher (lower) than normal temperatures and lower (higher) than normal rainfall, which are consistent with the results obtained from the composite analysis. Monsoon (ICMI) and ENSO (Niño3) are the climate modes explaining mostly the annual yields in Peninsular Malaysia (Fig. 6e). Finally, temperatures (minimum and maximum) during boreal spring have a strong control on the quantity of annual yields (Fig. 6c) in Sabah/Sarawak. The role of rainfall in the boreal fall of the year before the yield is also not negligible. Nevertheless, ENSO control on annual yields (Fig. 6) in Sabah/Sarawak is not that important compared to whole Malaysia and Peninsular Malaysia.

Statistical models based on linear regression are trained on the annual yields during the period 1987–2014. For each region, a model is constructed using actual values of local atmospheric variables (Model 1). Then, indices of large-scale climate phenomena (i.e. suppliers of the interannual variability) are added to the model (Model 2) to test the effects of both local and large-scale information. Local indices include effect of large-scale climate, but in a non-linear way that would be difficult to interpret and discuss. So, global parameters are directly incorporated in the study for the ease of explanations of linear relationships and to improve the model. Table 1 summarizes the local and large-scale variables included in statistical models in each region. For whole Malaysia, Model 1 (Equation 1) is based on local precipitation taken in Jul_−1_ (i.e. in July the year before the yields are considered):

1 $${\rm{Yield}}=13.8+0.0259\,{{\rm{PRCP}}}_{Ju{l}_{-1}}+{\rm{\varepsilon }}\,$$

**Table 1:** Statistical linear models defined in Malaysia, Peninsular Malaysia and Sabah/Sarawak.

Region	Model	Equation
Malaysia	1	$13.8+0.0259\,{{\rm{PRCP}}}_{Ju{l}_{-1}}$
	2	$15.7+0.0158\,{{\rm{PRCP}}}_{Ju{l}_{-1}}-0.6780\,\text{Ni}{{\tilde{\rm n}}}{\rm{o}}{3}_{Ma{r}_{0}}-0.999\,{{\rm{NNI}}}_{Ju{l}_{0}}$
Peninsular Malaysia	1	$47.7949-0.9606\,{\rm{TX}}2{{\rm{M}}}_{Ap{r}_{0}}$
	2	$41.0307-0.7398\,{\rm{TX}}2{{\rm{M}}}_{Ap{r}_{0}}+0.3025\,{{\rm{ICMI}}}_{Ma{r}_{0}}$
Sabah/Sarawak	1	$-29.8+0.0252\,{{\rm{PRCP}}}_{Ju{n}_{-1}}+1.5\,{\rm{TX}}2{{\rm{M}}}_{De{c}_{-2}}$
	2	$-24.8+0.0232\,{{\rm{PRCP}}}_{Ju{n}_{-1}}+1.34\,{\rm{TX}}2{{\rm{M}}}_{De{c}_{-2}}\,\mbox{--}\,0.541\,\text{Ni}{{\tilde{\rm n}}}{\rm{o}}{3}_{Ma{r}_{0}}$

Model 1 only includes local variables, while Model 2 adds remote climate modes to Model 1.

Annual yields estimated by Model 1 are slightly overestimated as the mean error (ME) is negative (Table 2), while the mean absolute error (MAE) is 0.5 Mg ha^−1^ and the percentage error of this model is around 3%. Model 1 explains 1/3 of the total variance (*r* = 0.627, R^2^ = 39.4%).. Other measurements of accuracy are given in Table 2. Model 2 (Equation 2) is the association of Model 1, ENSO (Mar_0_) and Ningaloo Niño/Niña (Jul_0_):

2 $${\rm{Yield}}=15.7+0.0158\,{{\rm{PRCP}}}_{Ju{l}_{-1}}-0.6780\,\text{Ni}{{\tilde{\rm n}}}{\rm{o}}{3}_{Ma{r}_{0}}-0.999\,{{\rm{NNI}}}_{Ju{l}_{0}}+\varepsilon $$

**Table 2:** Comparison of accuracy measurements between Model 1 (local variables) and Model 2 (adds remote climate modes to Model 1) in Malaysia, Peninsular Malaysia and Sabah/Sarawak.

Region	Model	ME	RMSE	MAE	MPE	MAPE	MASE	ACF1
Malaysia	1	−1.79e–13	0.699	0.544	−0.149	2.97	0.529	−0.234
	2	4.10e–14	0.421	0.331	−0.050	1.76	0.322	−0.404
Peninsular Malaysia	1	7.57e–14	0.662	0.553	−0.1280	3.0	0.476	−0.0941
	2	6.67e–14	0.545	0.426	−0.0858	2.3	0.367	−0.1100
Sabah/Sarawak	1	3.02e–13	1.010	0.805	−0.342	4.55	0.780	−0.0115
	2	3.45e–13	0.916	0.701	−0.281	3.94	0.679	0.2250

ME is the Mean Error (Mg ha^−1^), RMSE is the Root Mean Square Error (Mg ha^−1^), MAE is the Mean Absolute Error (Mg ha^−1^), MPE is the Mean Percentage Error (%), MAPE is the Mean Absolute Percentage Error (%), MASE is the Mean Absolute Scaled Error and ACF1 is the autocorrelation of errors at lag 1. Values are estimated from bootstrap (10,000 permutations).

**Figure 7:**
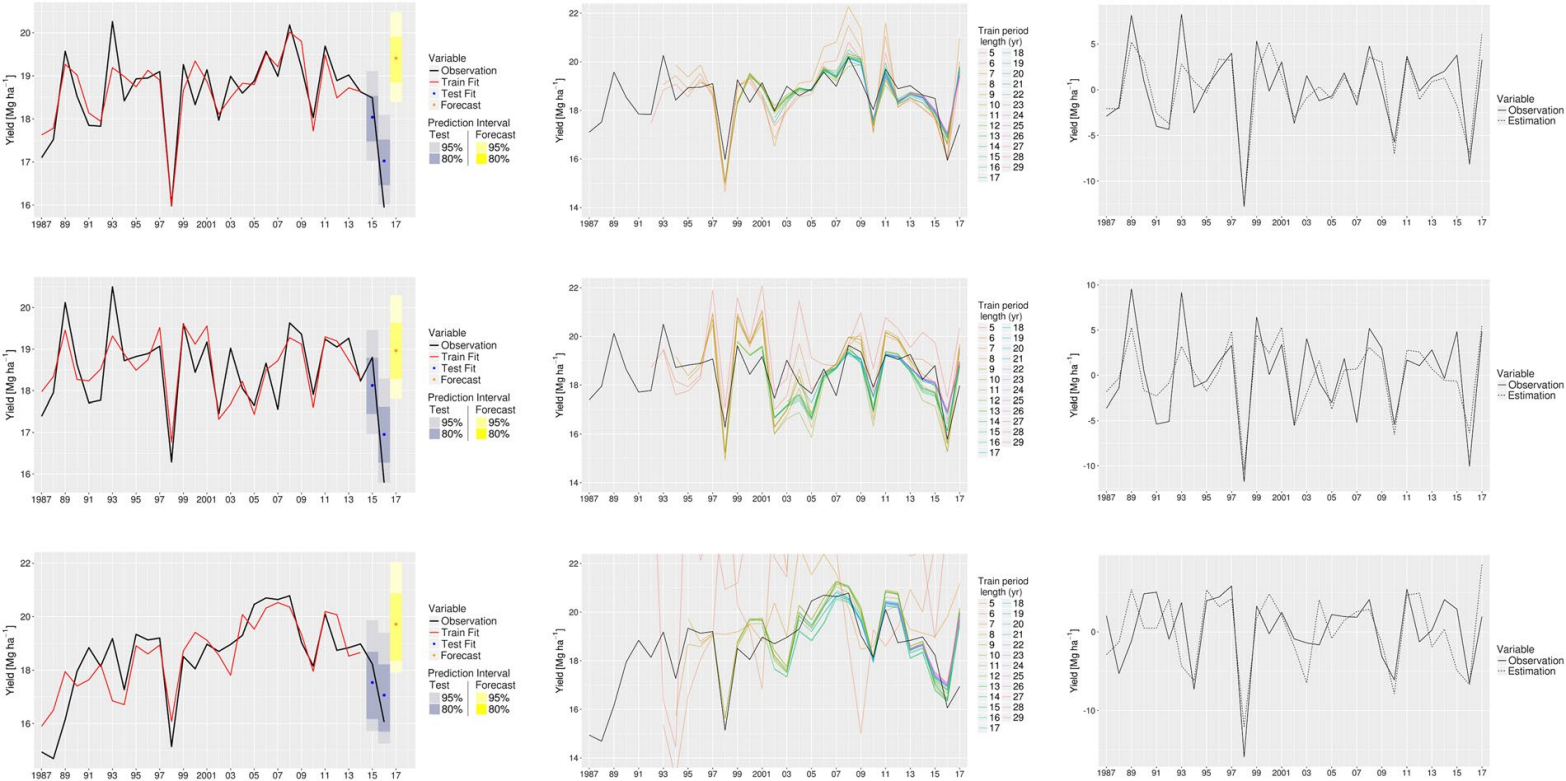
Left column: observation (black line, 1987–2016) and simulated annual yields by Model 2 for the training period (red line, 1987–2014), the test years (blue dot, 2015 and 2016) and forecast (yellow dot, 2017) of annual FFB yields in (**a**) Malaysia, (**d**) Peninsular Malaysia and (**g**) Sabah/Sarawak. Black line is the observed yields; blue shadings are prediction intervals for test and yellow shadings are prediction intervals for forecast. Central column: stability of Model 2 for (**b**) Malaysia, (**e**) Peninsular Malaysia and (**h**) Sabah/Sarawak. Black line is the reference and each colored line is a simulation with regression coefficients estimated with five years (5), six years (6) and so on, up to twenty-nine years (29). Right column: interannual variation of yields (in percentage) for (**c**) Malaysia, (**f**) Peninsular Malaysia and (**i**) Sabah/Sarawak. Observation is the black continuous line and simulation by Model 2 is the black dotted line. The figure was generated by R software version 3.4.1 (https://www.R-project.org/) with package “*ggplot2*” version 2.2.1.

According to ME (4.10e-14 Mg ha^−1^), MAE (0.3 Mg ha^−1^) and percentage error (1.8%), Model 2 generates less errors than that of Model 1 for simulating the observed FFB yields. This model also performs better in simulating the interannual variability of annual yields (*r* = 0.883, R^2^ = 78%). Complementary accuracy measurements of the association can be found in Table 2. Also, an analysis of variance^56^ is performed between Model 1 and Model 2, to estimate how Model 2 performs better than Model 1. The explained variances are given in Table 3. The *F*-ratio (F) shows an improvement of the model introducing large-scale indices, as the level of significance is close to 100%. The simulated annual yields by Model 2 for the period 1987–2014 are presented in Fig. 7a. Also, 2015 and 2016 are taken a test case to evaluate the accuracy of the selected model. The calculated values for these years are presented together with the forecast for the year 2017. This gives particularly accurate results on training and test periods. And we expect the forecast for the year 2017 to be successful. The stability of the model is also tested and result is shown in Fig. 7b. Starting with five years training and by adding one year at each iteration, it is found that the stability of Model 2 is very strong, as seen in the small difference between colored lines (simulated yields) and black lines (observed yields). This indicates that the variables used to construct Model 2 are very effective and stable in relationships during the period 1987–2014. Finally, the comparison between simulated and observed yields (Fig. 7c) does not show any difference in the calculated annual rate of variations.

**Table 3:** Analysis of variance between linear regressions models for Malaysia, Peninsular Malaysia and Sabah/Sarawak.

Region	Model	Res.Df	RSS	Df	Sum of Sq	F	Pr( > F)
Malaysia	1	26	13.6773				
	2	24	4.9542	2	8.7231	21.129	5.102e–06
Peninsular Malaysia	1	26	12.258				
	2	25	8.324	1	3.9342	11.816	0.002065
Sabah/Sarawak	1	26	45.918				
	2	24	23.491	2	22.427	11.456	0.0003214

Model 1 only includes local variable, while Model 2 adds remote climate modes to Model 1. Res.Df is the residual degrees of freedom, RSS is the residual sum of squares, Df is the degree of freedom, Sum of Sq is the difference in RSS, F is the value of the F-ratio and Pr( > F) is the associated *p*-value.

In Peninsular Malaysia, maximum temperature at 2-m height (Apr_0_) is the basis of Model 1 (Equation 3)

3 $${\rm{Yield}}=47.7949-0.9606\,{\rm{TX}}2{{\rm{M}}}_{Ap{r}_{0}}+\varepsilon $$

The level of errors produced by this model is comparable to that of Model 1 for the whole Malaysia, i.e. very small ME (with a slight underestimation), MAE around 0.5 Mg ha^−1^ and percentage error of 3% (see Table 2 for other accuracy measurements). Model 1 simulates about 50% of the total variance of observed annual yields (*r* = 0.691, R^2^ = 47.7%). The monsoon index (Mar_0_) is added to this model to obtain Model 2 (Equation 4):

4 $${\rm{Yield}}=41.0307-0.7398\,{\rm{TX}}2{{\rm{M}}}_{Ap{r}_{0}}+0.3025\,{{\rm{ICMI}}}_{Ma{r}_{0}}+\varepsilon $$

Once again, Fisher test shows a real improvement of the model (level of significance close to 99.9%) when local and large-scale information are combined together (Table 3). In comparison (Table 2), Model 2 actually gives better results than Model 1 in simulating yields, reducing ME (6.67e-14 Mg ha^−1^), MAE (0.4 Mg ha^−1^) and percentage error (2.3%). Simulation results are also realistic for the period 1987–2014 (Fig. 7d,f), reproducing 64.5% of the total variance (*r* = 0.803). But the model appears to be less stable when period of the regression coefficients is changed (Fig. 7e).

In Sabah/Sarawak, Model 1 (Equation 5) is constructed using local precipitation in Jun_−1_ and maximum temperatures at 2-m height in Dec_−2_:

5 $${\rm{Yield}}=-29.8+0.0252\,{{\rm{PRCP}}}_{Ju{n}_{-1}}+1.5\,{\rm{TX}}2{{\rm{M}}}_{De{c}_{-2}}+\varepsilon $$

The errors produced by Model 1 in this region are higher (MAE of 0.8 Mg ha^−1^ and percentage error of 4.5%) than that in other geographical entities, while remaining reasonably low. The variance explained by the model is quite high (*r* = 0.776, R^2^ = 60.2%). Model 2 (Equation 6) is then constructed including Niño3 index (Mar_0_):

6 $${\rm{Yield}}=-24.8+0.0232\,{{\rm{PRCP}}}_{Ju{n}_{-1}}+1.34\,{\rm{TX}}2{{\rm{M}}}_{De{c}_{-2}}-0.541\,\text{Ni}{{\tilde{\rm n}}}{\rm{o}}{3}_{Ma{r}_{0}}+\varepsilon $$

Inclusion of ENSO index in Model 1 increases the ability to describe the interannual variability of FFB yields (Table 3) by Model 2. The accuracy of Model 2 is higher (Table 2) also; the errors generated are reduced to 0.7 Mg ha^−1^ (MAE) and the percentage error becomes less than 4%. However, among three regions, the statistical model based on local variables and large-scale indices is less accurate (*r* = 0.819, R^2^ = 67%). While the trend is captured (Fig. 7g), the stability of the model is very low when utilizing only a few years for training (Fig. 7h). It is only after including a long training period (about twenty years of data) the model tends to stabilize and the interannual variation rate is reproduced to some extent (Fig. 7i).

The models for the three areas are based on different variables accounting for the different climatic conditions in different parts of Malaysia. Thus, the use of maximum temperature in Peninsular Malaysia illustrates the continental influence on this area while use of precipitation in the Sabah/Sarawak model illustrates the oceanic influence.

## Conclusion

In this study, it is found that teleconnections arising from the equatorial Pacific Ocean and the eastern Indian Ocean play an important role on local water stress and temperatures in Malaysia, which in turn control the yearly production of fresh fruit bunches on country as well as regional scales. The results show association of palm yields with ENSO, ENSO Modoki and some other regional variations. Particularly, spring rainfall of yield year decreases together with an increase in atmospheric temperatures following an El Niño occurrence during the previous winter. Local water stress is then enhanced, favoring a reduction in total yields. Also, an interesting signal is seen in the region of the Ningaloo Niño/Niña– the newly discovered mode of climate variations off Western Australia. This needs to be further explored.

By combining the effect of local climate with the large-scale climate modes, such as ENSO, statistical palm yield prediction models are developed here with a reasonable success in terms of stability and reliability. While such an approach may be sensitive to climate regime shift and subsequent changes in the local/large-scale relationships, it is not a concern for the analysis of results on a fixed time-frame.

Further research should also be performed at finer regional scales over Malaysia (for example at a provincial scale), but such fine-scale data generally lack the accuracy and it may be difficult to distinguish a meaningful signal from the background noise. For this purpose, dynamical downscaling can be used to introduce more realistic spatial variability taking into account the effect of local features, such as the orography and the vegetation on the local weather and climate. By using biased corrected and downscaled seasonal climate forecasts, we can stretch the forecast limits of the statistical model. Further, the seasonal time scale is also interesting and needs further detailed and extensive research. It will be necessary to study seasonal variations in the influences arising from remote phenomena such as ENSO and Ningaloo Niño/Niña and how those affect the physiology of palm trees. Considering the potential advantage, this research is expected also to contribute to Indonesia, the leading producer of oil palm.

## Methods

The annual yields of fresh fruit bunches are taken from the Malaysian Palm Oil Board website^41^, for the period of January 1987-December 2015. This period is chosen as reference period for all dataset used in this paper. To characterize the interannual variability of FFB yields, the year-to-year variation rate is used, following the method described earlier^42^. This rate is meant to capture only the natural variability, removing other factors, such as human action. In this study, we adopted the calculation based on a locally weighted smoothing (loess)^57^, with a degree of smoothing of 0.32 and a degree 1 for polynomials. Results are similar to those calculated using a 5-year running mean, as already highlighted by the earlier study^42^.

The Extended Reconstructed Sea Surface Temperature (ERSST) version 4^58–60^ with a 2° × 2° horizontal resolution, and for the period 1984–2015, is used.

The Global Precipitation Climatology Centre (GPCC) data^61^ are used for monthly precipitation, with a 1° × 1° horizontal resolution, and covering the period 1984–2015. GPCC Precipitation data provided by the NOAA/OAR/ESRL PSD, Boulder, Colorado, USA, from their Web site at http://www.esrl.noaa.gov/psd/.

The monthly Niño3 Index is provided by CPC/NOAA, College Park, Maryland, USA, from their Web site at http://www.cpc.ncep.noaa.gov/data/indices/. The monthly El Niño Modoki Index is provided by JAMSTEC, Yokohama, Japan, from its Web site http://www.jamstec.go.jp/frcgc/research/d1/iod/enmodoki_home_s.html.en.

The Ningaloo Niño/Niña Index was calculated following Kataoka *et al*. definition^48^. The monthly Indian Ocean Dipole Index is provided by NOAA from its Web site http://stateoftheocean.osmc.noaa.gov/sur/ind/dmi.php.

A monsoon index for the region study is constructed following Tsai *et al*.^62^. The index is the difference between the area-averaged 850-hPa zonal wind anomalies in the domain 85°E-95°E, 7.5°N-12.5°N and the area-averaged 850-hPa meridional wind anomalies in the domain 120°E-122.5°E, 0-8.75°N. This allows the seasonal change in the wind direction over the country to be captured.

Monthly mean, minimum and maximum air temperature at 2 meters, and surface net solar radiation are directly taken from the European Reanalysis Interim (ERA-Interim) dataset^63^, with a 0.75° × 0.75° horizontal resolution and for the period 1984–2015. Data are resampled to match the GPCC grid using a bilinear interpolation. The relative humidity at 2 meters (Equation 7) is not directly available and is derived from the saturation water vapor pressure of both the temperature and the dew point temperature at 2-meter height (Equation 8), following ECMWF recommendation^64^:

7 $$RH=100\times \frac{{e}_{sat}(Td)}{{e}_{sat}(T)}$$

*e*_*sat*_() = saturation vapor pressure (Pa), *Td* = dew point temperature at 2 meters (K), *T* = air temperature at 2 meters(K).

8 $${e}_{sat}(T)={a}_{1}exp\{{a}_{3}(\frac{T-{T}_{0}}{T-{a}_{4}})\}$$

*T* = temperature at 2 meters (K), *a*_1_ = 6.1121 (hPa), *a*_3_ = 17.502, *a*_4_ = 32.19 (K), *T*_0_ = 273.16 (K).

The daily potential evapotranspiration is calculated following the Penman-Monteith FAO56 Reference Crop evapotranspiration^65^ for short reference^66^ (Equation 9) using the ERA-Interim data (air temperature, relative humidity, dew point temperature, solar radiation, wind speed, total cloud cover). Wind speed at 2 meters are derived from wind speed at 10 meters following Equation 47 in Allen *et al*.^65^. Monthly means are calculated from the daily values.

9 $$E{T}_{0}=\frac{0.408{\rm{\Delta }}({R}_{n}-G)+\gamma \frac{900}{T+273}{u}_{2}({e}_{s}-{e}_{a})}{{\rm{\Delta }}+\gamma (1+0.34{u}_{2})}$$

*ET*_0_ = standardized reference crop evapotranspiration (mm d^−1^), *R*_*n*_ = calculated net radiation at the crop surface (MJ m^−2^ d^−1^), *G* = soil heat flux density at the soil surface (MJ m^−2^ d^−1^), *T* = mean daily air temperature near the surface (°C), *u*_2_ = mean daily wind speed at 2-m height (m s^−1^), *e*_*s*_ = saturation vapor pressure near the surface (kPa), *e*_*a*_ = mean actual vapor pressure near the surface (kPa), *Δ* = slope of the saturation vapor pressure-temperature curve (kPa °C^−1^), *γ* = psychrometric constant (kPa °C^−1^), 0.408 = numerator constant (m^2^ mm MJ^−1^), 900 = numerator constant (K mm s^3^ Mg^−1^ d^−1^), 0.34 = denominator constant (s m^−1^).

The vertically integrated moisture flux (Equation 10, in kg m^−1^ s^−1^) is calculated utilizing the surface pressure (${\hat{p}}_{s}$), the specific humidity (*q*) and the vector wind components (*W*, either zonal or meridional).

10 $${\int }_{0}^{{\hat{p}}_{s}}(Wq){\delta }_{p}/g$$

The mass-weighted sum is calculated between the surface and the 250 hPa pressure level, which correspond to 21 levels in ERA-Interim and 20 pressure differences (𝛿_*p*_). The gravity of Earth (*g*) is fixed to 9.807 m s^−2^.

For rainfall, temperature, relative humidity, potential evapotranspiration and solar radiation, only grid points over palm oil plantation (as estimated in 2011) are kept. The selection is based on Fig. 1 in Koh *et al*.^8^. This gives a total of 106 grid points, covering both Peninsular Malaysia and Sabah/Sarawak. It has to be noted that cumulative land area of palm oil plantations between 1987 and 2011 increased, meaning that in the first years of the study period, many grid points are not covered by oil palms, possibly introducing a bias in the model. However, considering the size of the regions, the signal (i.e. the relationship climate/total yields) is greater than the noise (i.e. absence of oil palms).

Anomalies to the 1982–2011 period mean are calculated and the linear trend is removed from all dataset using a least-square fit. Three-month-running means are adopted for all indices to minimize the intraseasonal variations. The confidence level in Fig. 6 is based on a random-phase test^67^. For this test, a large number of random time series (here 10,000 simulations) that have the same power spectrum as the original time series, but with random phases, are generated. The statistical significance is defined as the proportion of random correlations (coming from simulations) lower (if the observed correlation is negative) or higher (if the observed correlation is positive) than the observed correlation.

The partial correlation is used to remove the influence of the ENSO and estimate the direct interaction between annual FFB yields and climate modes. The calculation is based on matrix inversion^68^ as the partial correlations are the negative standardized covariances.

Composites analysis is a useful tool to identify conditions observed during specific states of the climate. This statistical method involves the computation of the composite mean over selected time steps, for example the set of years with low FFB yields in Malaysia. Composite analysis can be represented by composited time series (Fig. 2) or composited maps (Figs 3–5). The statistical significance of composite analysis in Fig. 2 and Figs S1 and S2 (H_1_: composite mean for years with low FFB is different from composite mean for years with high FFB) is based on a two-sided Student’s *t*-test^69,70^ with 10,000 permutations. The statistical significance of composite analysis in Figs 3–5 (H_1_: composite mean for years with low FFB is different from composite mean for years with high FFB) is based on a two-sided Student’s *t*-test for SST anomalies and on an Hotelling’s T2 test^71,72^ for the vertically integrated moisture flux anomalies, both with 5,000 permutations.

The accuracy of fitted model (i.e. *goodness of fit*) is estimated using different common measurements^73,74^. Provided values are the difference between the estimation and the observation or Mean Error (ME), the sample standard deviation of the differences between predicted values and observed values or Root Mean Square Error (RMSE), an index of proximity of prediction to eventual outcome or Mean Absolute Error (MAE), the computed average of percentage errors by which forecasts of a model differ from actual values of the quantity being forecast or Mean Percentage Error (MPE), the forecast bias or Mean Absolute Percentage Error (MAPE), the forecast accuracy or Mean Absolute Scaled Error (MASE) and the autocorrelation of errors at lag 1 (ACF1). The smaller the values, the better the fit. These values are estimated from bootstrap (10,000 permutations). However, as the time series used to develop and verify statistical models are quite short, the accuracy of the fitted models might be highly variable. In order to test the stability of the model, variables are first chosen for the period 1987–2014 and are fixed. Second, the coefficients associated to the model are calculated for first five years (i.e. 1987–1991) and simulated yields for the remaining period (1992–2016), then coefficients are estimated for six first years (i.e. 1987–1992) and yields estimated for the period 1993–2016, and so on, up to the 1987–2014 (train) / 2015–2017 (test and forecast) periods. Observed and simulated yields are then plotted together. If the simulated yields don’t dramatically deviate from the observation, the model is considered as stable. The accuracy of forecasts is estimated by calculating the prediction intervals^75^, i.e. the probability for the observed annual yield to lie within the interval. The selected probabilities are 80% and 95%.

All calculations and graphics are made using the R project for statistical computing version 3.4.1^76^, with the help of packages “*forecast*” version 8.1 (http://github.com/robjhyndman/forecast), “*Hotelling*” version 1.0–4 (https://CRAN.R-project.org/package=Hotelling), “*Evapotranspiration*” version 1.10 (https://CRAN.R-project.org/package=Evapotranspiration) and “*ppcor*” version 1.1 (https://CRAN.R-project.org/package=ppcor) for calculations, and packages “*rasterVis*” version 0.41 (http://oscarperpinan.github.io/rastervis/) and “*ggplot2*” version 2.2.1 (http://ggplot2.org) for graphics.

## Electronic supplementary material

Supplementary Information
