# Supplementary material for: Climate Based Predictability of Oil Palm Tree Yield in Malaysia
Source: Sci Rep. 2018 Feb 2;8:2271. doi: 10.1038/s41598-018-20298-0 (PMC5797103; doi:10.1038/s41598-018-20298-0)
Supplement: Supplementary file 1 — Supplementary Information [file 41598_2018_20298_MOESM1_ESM.doc]

**Supplementary Information to manuscript "Climate Based Predictability of Oil Palm Tree Yield in Malaysia"**

**Pascal Oettli^1,*^, Swadhin K. Behera^1^, Toshio Yamagata^1^**

Supplementary Figure S1. Composite analysis of deviations from the mean for a) rainfall, b) maximum temperature at 2-m height, c) minimum temperature at 2-m height, d) relative humidity at 2-m height, e) solar radiation and f) potential evapotranspiration for Peninsular Malaysia. Vertical bars denote the standard error of the mean. Shaded areas denote stress-sensitive periods considering the peak of fruits harvesting in September-October, i.e. sex determination (dark grey), inflorescence abortion (grey) and bunch failure (light grey). Significant differences between means at 95% level (according to two-sided Student’s *t*-test with 10,000 permutations) are showed by black dots. The figure was generated by R software version 3.4.1 with package “*ggplot2*” version 2.2.1.

Supplementary Figure S2. Composite analysis of deviations from the mean for a) rainfall, b) maximum temperature at 2-m height, c) minimum temperature at 2-m height, d) relative humidity at 2-m height, e) solar radiation and f) potential evapotranspiration for Sabah/Sarawak. Vertical bars denote the standard error of the mean. Shaded areas denote stress-sensitive periods considering the peak of fruits harvesting in September-October, i.e. sex determination (dark grey), inflorescence abortion (grey) and bunch failure (light grey). Significant differences between means at 95% level (according to two-sided Student’s *t*-test with 10,000 permutations) are showed by black dots. The figure was generated by R software version 3.4.1 with package “*ggplot2*” version 2.2.1.


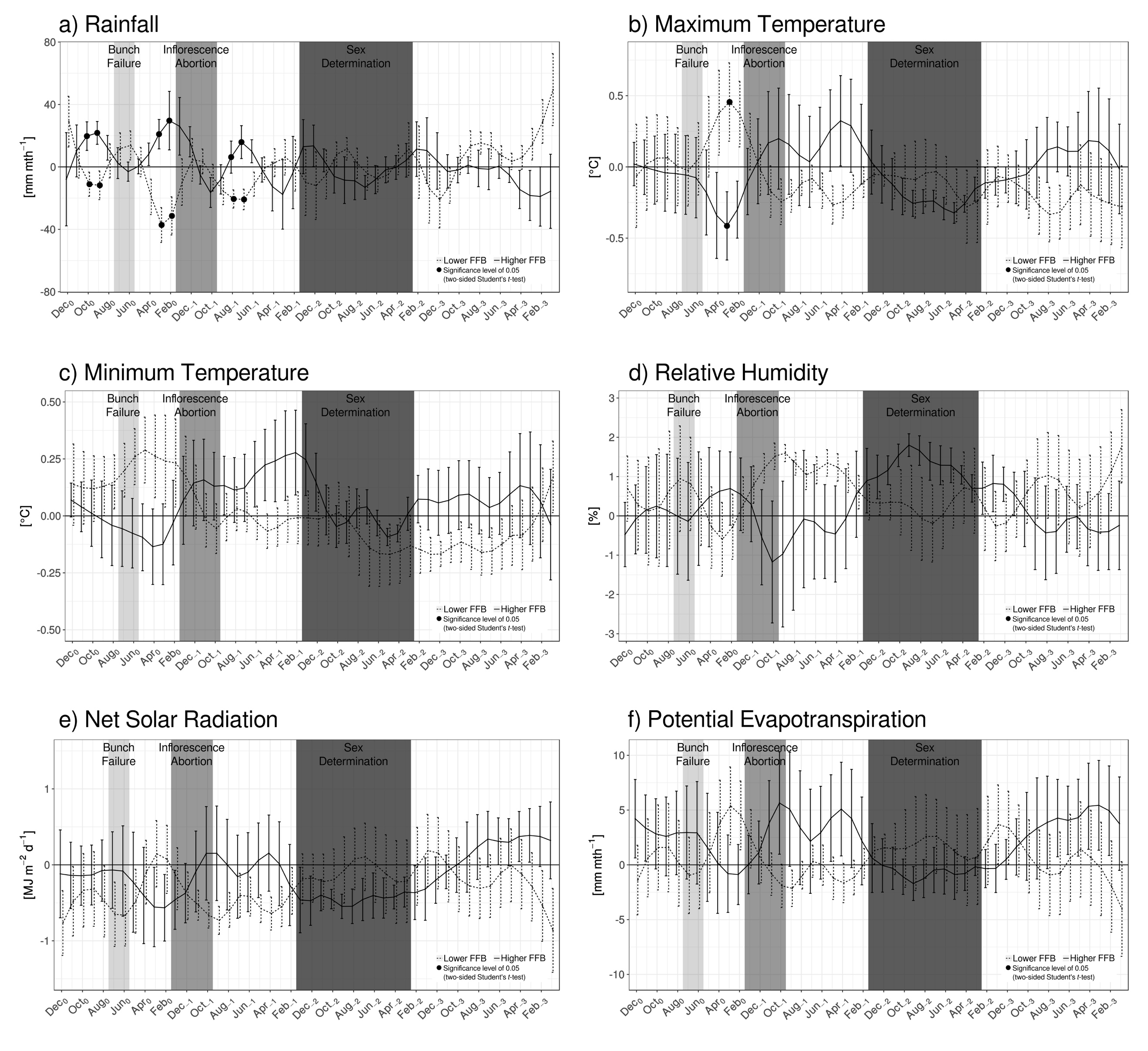


**Supplementary Figure S1.** Composite analysis of deviations from the mean for a) rainfall, b) maximum temperature at 2-m height, c) minimum temperature at 2-m height, d) relative humidity at 2-m height, e) solar radiation and f) potential evapotranspiration for Peninsular Malaysia. Vertical bars denote the standard error of the mean. Shaded areas denote stress-sensitive periods considering the peak of fruits harvesting in September-October, i.e. sex determination (dark grey), inflorescence abortion (grey) and bunch failure (light grey). Significant differences between means at 95% level (according to two-sided Student’s t-test with 10,000 permutations) are showed by black dots. The figure was generated by R software version 3.4.1 with package “*ggplot2*” version 2.2.1.


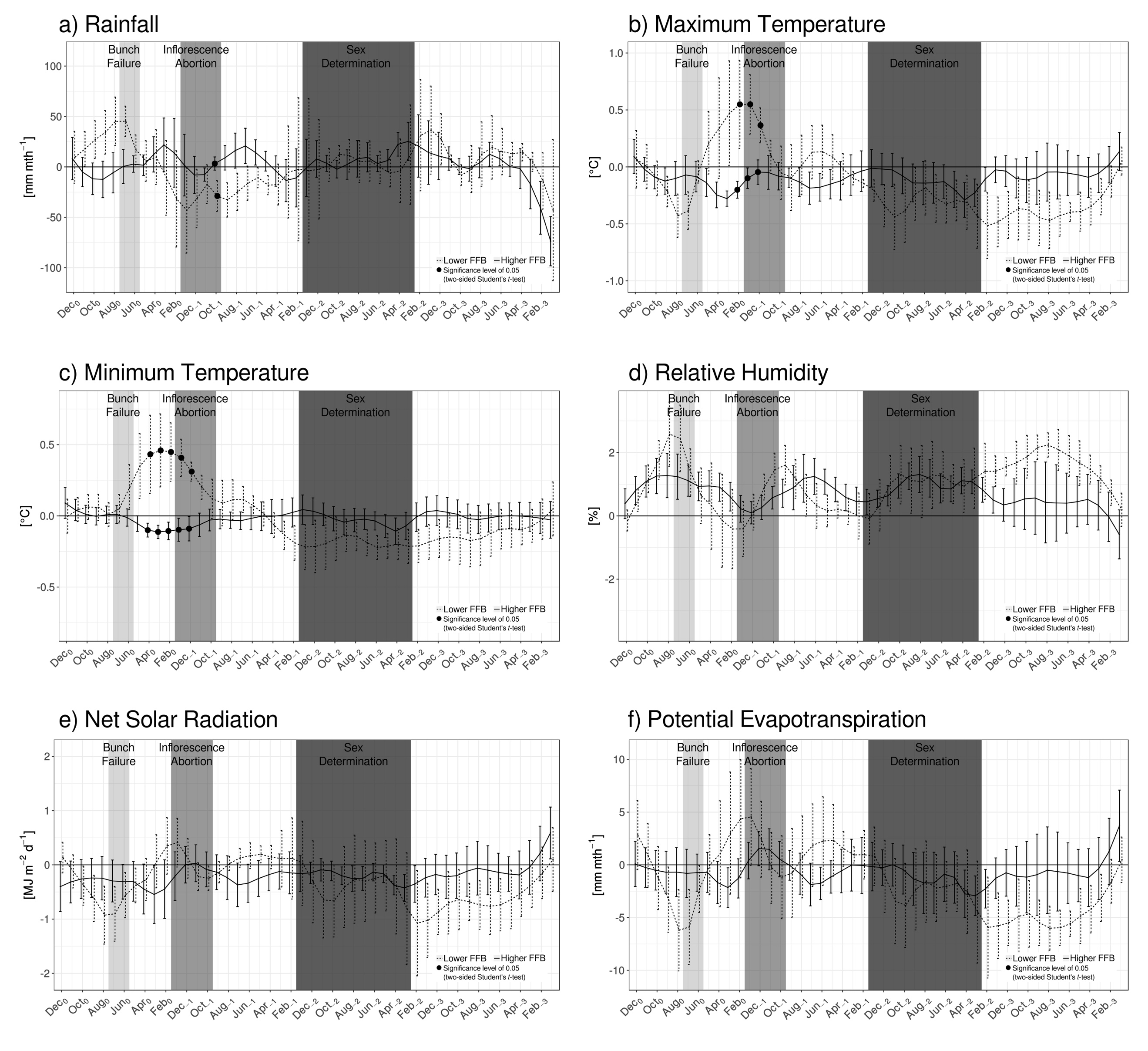


**Supplementary Figure S2:** Composite analysis of deviations from the mean for a) rainfall, b) maximum temperature at 2-m height, c) minimum temperature at 2-m height, d) relative humidity at 2-m height, e) solar radiation and f) potential evapotranspiration for Sabah/Sarawak. Vertical bars denote the standard error of the mean. Shaded areas denote stress-sensitive periods considering the peak of fruits harvesting in September-October, i.e. sex determination (dark grey), inflorescence abortion (grey) and bunch failure (light grey). Significant differences between means at 95% level (according to two-sided Student’s t-test with 10,000 permutations) are showed by black dots. The figure was generated by R software version 3.4.1 with package “*ggplot2*” version 2.2.1.
